# On the Use of Normalized Compression Distances for Image Similarity Detection

**DOI:** 10.3390/e20020099

**Published:** 2018-01-31

**Authors:** Dinu Coltuc, Mihai Datcu, Daniela Coltuc

**Affiliations:** 1 Faculty of Electrical Engineering, Electronics and Information Technology, Valahia University of Targoviste, Târgoviște 130024, Romania; 2 Remote Sensing Technology Institute, German Aerospace Center (DLR), Germany and Research Centre for Spatial Information, Politehnica University of Bucharest, București 060042, Romania; 3 Research Centre for Spatial Information, Politehnica University of Bucharest, București 060042, Romania

**Keywords:** normalized information distance, normalized compression distance, NCD feature vectors, lossless compression, image similarity, robust similarity

## Abstract

This paper investigates the usefulness of the normalized compression distance (NCD) for image similarity detection. Instead of the direct NCD between images, the paper considers the correlation between NCD based feature vectors extracted for each image. The vectors are derived by computing the NCD between the original image and sequences of translated (rotated) versions. Feature vectors for simple transforms (circular translations on horizontal, vertical, diagonal directions and rotations around image center) and several standard compressors are generated and tested in a very simple experiment of similarity detection between the original image and two filtered versions (median and moving average). The promising vector configurations (geometric transform, lossless compressor) are further tested for similarity detection on the 24 images of the Kodak set subject to some common image processing. While the direct computation of NCD fails to detect image similarity even in the case of simple median and moving average filtering in 3 × 3 windows, for certain transforms and compressors, the proposed approach appears to provide robustness at similarity detection against smoothing, lossy compression, contrast enhancement, noise addition and some robustness against geometrical transforms (scaling, cropping and rotation).

## 1. Introduction

The Kolmogorov complexity defines the information content of a single digital data object as the length of the shortest computer program that output it. Furthermore, the similarity between two digital objects can be measured by the information required to transform each object into the other. This elegant theory is brought to life by approximating the non-computable Kolmogorov complexity with the size of the lossless compressed data. In this framework, for computing the theoretical information distances, several distances have been defined. The prominent example is the normalized compression distance (NCD), a parameter-free, feature-free, alignment-free, similarity metric based on compression [1,2]. NCD provides a computable version of the normalized information distance (NID) [1]. Besides NCD, other compression based approaches for data similarity have been proposed as, for instance, the PRDC (Pattern Representation using Data Compression) [3].

NCD and, generally, compression based distances, have already been used in several applications. One can mention the automatic construction of the phylogeny tree based on whole mitochondrial genomes and a completely automatic construction of a language tree [1,2], classification of DNA sequences [4], music classification [5,6], data mining [7], authorship attribution [8], image registration [9], optical character recognition [10], similarity between web pages for phishing detection [11], automatic classification of intracardiac electrograms [12], etc.

The generality of compression based distances is very attractive. Theoretically, such distance could be used “without changes to different areas and even to collections of objects taken from different areas” to “automatically zooms in on the dominant similarity aspect between every two objects” [2]. In [2], it is said that “all data are created equal”. In fact, some data are more equal than other. This is the case of digital images. Obviously, image data are different from alphanumeric text, time series, computer programs, etc. Thus, images are intrinsically two-dimensional structures, while the other data mentioned above are one-dimensional. A more subtle aspect is related to the fact that, usually, when images are compared, one should deal with some robustness against specific processing like contrast enhancement, filtering, lossy compression, scaling, cropping, small rotations, etc. In other words, one should check the identity not of the numerical data, but of the image content.

As discussed above, compression based metrics have already been used with digital images (see [9,10], etc.). NCD is known to have a certain robustness against noise [13]. On the other hand, the robustness against image processing and geometric transforms is low. For instance, in [14], the results for a set of common image processing algorithms (addition of noise, median filtering, histogram equalization, watermarking, etc.) are presented. The similarity between the original and the processed versions cannot be detected. Thus, instead of taking small values as expected, the NCD between the original and the processed versions can take large values. In [15], the robustness against scaling, rotation and translation has been investigated. The authors have tested several compressors and image formats for the computation of NCD. From their tests, it clearly appears that the robustness of NCD against geometric transforms is low. The conclusion of the authors is that NCD cannot be applied without taking care of the image format or of the compression software.

Generally, the compressors used by NCD deal with sequential files and, consequently, images should be converted to one-dimensional sequences before the computation of the distance. In [16], the conversion from 2D to 1D is investigated. The paper shows that the results of the NCD depend on the scanning method used to convert the images. The use of multiple linearizations for one comparison pair may improve the results. Thus, the Hilbert–Peano scanning may capture better similarity among rotated copies than other forms of linearizations. Furthermore, depending on the orientation of the image features, the column-by-column scanning may provide better results than the row-by-row one.

The limited efficiency of NCD with ready-made lossless compressors when dealing with images has determined the researchers to look for other solutions. Thus, in [17], the authors have built their own encoder for grayscale images inspired by JBIG lossless image compression standard ISO/IEC standard 11544. The encoder is based on finite-context modeling, but, different from JBIG, it uses simultaneous multiple contexts combined using a mixture model. Then, instead of working on planes, it works on entire pixels. In order to limit the memory resources, the image is first lossy quantized to four bits. Tested in a content based image retrieval application, the normalized compression distances with the proposed encoder outperformed the ones with some lossless compressors especially developed for images (lossless JPEG2000, lossless JPEG, JBIG).

An innovative solution is the Campana–Keogh distance, CK-1, [18,19], based on the lossy video compressor MPEG1. The use of a video compressor instead of a standard lossless compressor takes advantage of the mechanisms developed for video sequences, like motion compensation, interframe coding, etc. The four video sequences of two frames are created by combining the two images under consideration and the distance between the images follows as the ratio between the sum of the sizes of the MPEG compressed cross-sequences divided by the sum of the sizes of the remaining sequences. The CK-1 distance was initially used with texture images [18]. In order to provide some robustness against contrast enhancement, the authors have considered the normalization of the images before CK-1 distance computation [19]. Furthermore, in order to solve rotations or translations, a distortion inversion scheme was used. More precisely, they compute the CK-1 distance between one fixed image and a sequence of rotations (translations) for the other image and look for the minimum CK-1 distance in order to detect similarity. One can also mention approaches based on dictionaries [20,21,22]. More precisely, first dictionaries are extracted from images and then distances between dictionaries are computed. Such approaches have been proposed for satellite image classification [20], image clustering, and image retrieval [21,22].

This paper investigates the usefulness of NCD with ready-made lossless compressors when dealing with images subject to common image processing like noise filtering, contrast enhancement, zoom, etc. While the direct use of NCD fails to detect image similarity in such cases, this paper shows that, in spite of this fragility, NCD can be used to extract robust features against such image processing tasks. More precisely, a set of simple geometric transforms are defined and, for each image, a feature vector is derived by taking the NCD between the original and the sequence of transformed images. Furthermore, instead of taking the NCD between two images, similarity is detected by computing the normalized correlation between the corresponding vectors.

The outline of the paper is as follows. The proposed approach is introduced in Section 2. A simple experiment for selecting feature vectors robust against image processing is presented in Section 3. Further results on the robustness of the selected feature vectors for image similarity detection are shown in Section 4. Finally, the conclusions are drawn in Section 5.

## 2. From Simple NCD to NCD Vectors

First, the theoretical model of the direct use of information distances for image similarity detection in the case of common image processing is investigated in Section 2.1 and, then, an improved solution using NCD feature vectors is proposed in Section 2.2.

Before going any further, we introduce some basic definitions (see [1,2]). Let *x* be a digital object, e.g., a binary string. We briefly remind readers that the Kolmogorov complexity of *x*, K(x), is the length of the shortest binary program that outputs *x*. Furthermore, the information distance between *x* and *y*, E(x,y), is the length of the shortest binary program that computes *y* from *x* and vice versa. One has:

(1) E(x,y)=max{K(x|y),K(y|x).}

The normalized information distance, NID, is defined as:

(2) $$NID\left(x,y\right)=\frac{\max\{K(x|y),K(y|x)\}}{\max\{K(x),K(y)\}}.$$

Computable approximations of information distances are obtained by replacing the Kolmogorov complexity with the size of the code provided by lossless compression. In this setting, NID is approximated by the normalized compression distance, NCD, defined as:

(3) $$NCD\left(x,y\right)=\frac{C(xy)-\min\{C(x),C(y)\}}{\max\{C(x),C(y)\}},$$

where C(x)(C(y)) represent the size (bits) of compressed *x* (*y*) and C(xy) the one of the joint compressed *x* and *y*.

### 2.1. Direct Information Distances between Images

Let *f* and *g* be two gray-level images. Let *A* be the shortest program that implements a specific image processing task and let $f^{*}=A\left(f\right)$ be the processed image. Let us first suppose that *A* is invertible and let $A^{-1}$ be the shortest program for the inverse algorithm. Let |A|, $|A^{-1}|$ be the sizes of the two computer programs. With the above notations, the information distance between the original and the processed image is given by Equation (1), $E\left(f,f^{*}\right)=\max\{|A|,|A^{-1}\left|\right\}$. Let us suppose that the task considered above is the upscaling with scale factor 2 by bilinear interpolation. Obviously, the computer program for upscaling has only a few programming lines. The inverse program, the simple subsampling without any low-pass filtering, is even shorter. Hence, $E(f,f^{*})$ should take a small value. Furthermore, the $NID(f,f^{*})$ (Equation (2)) needs the evaluation of K(f) and $K(f^{*}),$ respectively. The Kolmogorov complexity of natural images should depend on their content and on their size (number of pixels). It is reasonable to assume that the size of (the shortest) computer program that describes a common image processing algorithm is considerably shorter than the one that outputs the hundreds of thousands or even the millions of pixels of a natural image and, consequently, $NID(f,f^{*})$ is close to zero.

If, instead of the simple zoom, one considers a more complex image processing reversible algorithm (lossless compression, reversible watermarking, integral transforms, etc.), one expects to have the same behavior for $E(f,f^{*})$ and $NID(f,f^{*})$. Finally, let *f* and *g* be two different content natural images—for instance, different scenes acquired in different places. Obviously, knowing *f* does not provide any useful information to describe the pixels of *g* and one expects to have E(f,g) comparable to K(g). Hence, $E\left(f,f^{*}\right)<<E\left(f,g\right)$ and $0\approx NID\left(f,f^{*}\right)<<NID\left(f,g\right)$, i.e., for reversible image processing algorithms, the NID could detect the similarity between the original image *f* and the processed one, $f^{*}$.

The case of reversible algorithms is not very representative for image processing tasks. Generally, we expect to have not exact, but approximate results, i.e., $A^{-1}\left(f^{*}\right)\approx f$. This may be due to the rounding errors, to the fact that some operations involved by image processing algorithms are not invertible and so on. The case of approximate inversion can be easily transformed into exact inversion by adding a residual term:

(4) $$f=A^{-1}\left(f^{*}\right)+R\left(f,A^{-1}\left(f^{*}\right)\right),$$

where $R\left(f,A^{-1}\left(f^{*}\right)\right)=f-A^{-1}\left(f^{*}\right)$. The information distance becomes:

(5) $$E\left(f,f^{*}\right)=\max\{|A|,|A^{-1}\left|+K\left(R\left(f,A^{-1}\left(f^{*}\right)\right)\right).\right\}$$

The residual term can be approached as noise. We shall use the zero-order entropy to provide a rough estimate of the upper bound of its Kolmogorov complexity.

Let us next consider two examples of approximate inversion, namely of histogram equalization and of image smoothing by moving average. Histogram equalization is a well-known image enhancement technique that spreads image gray-levels over the entire scale and allocates an equal number of pixels to each gray-level. For human observers, this yields more balanced and better contrasted images [23]. Histogram equalization can merge some close gray-levels, and this operation is not invertible. A rough inversion of histogram equalization can be obtained by histogram specification [23], when the histogram to be specified is the histogram of the original image. Significantly better results can be obtained by using exact histogram specification [24]. While the classical equalization/specification by cumulative distribution function provides only approximate results, the exact histogram specification can separate among equal gray-level pixels in order to get exactly the desired histogram. We have shown that, for natural images, one can recover a very good approximation of the original by replacing the classical specification with the exact one. For instance, for the test image *Lena*, the PSNR of the recovered image is 58.45 dB [25]. The complexity of the classical histogram equalization algorithm is very low. The Matlab (Release 2015b, The MathWorks, Inc., Natick, MA, USA) script for our exact histogram specification has 70 lines and 1.161 kbytes (including comments), i.e., it is of negligible complexity, too. For *Lena*, the entropy of the residual image is of 0.31 bits. One can consider that the size of the residual term is bounded by 0.31NM, where N,M are the number of rows and columns of the image *f*. Obviously, $K\left(R\left(f,A^{-1}\left(f^{*}\right)\right)\right)>>\max\{|A|,|A^{-1}\left|\right\}$ and the corresponding $NID(f,f^{*})$ is not necessarily close to zero as in the case of reversible algorithms discussed above.

Let us briefly investigate the moving average filter in a window 3×3. In order to invert the smoothing, one can use inverse filtering, Wiener filtering, deconvolution by the Lucy–Richardson method, etc. For *Lena*, the best results have been obtained for the Lucy–Richardson method, namely a PSNR of 47.10 dB for the recovered image and an entropy of the residual image of 2.21 bits. Compared with histogram equalization, the upper bound of the residual (and, consequently, of the information distance $E(f,f^{*})$), increases. It should be noticed that 2.21 bits per pixel becomes comparable with the size of the natural gray-level images. Therefore, one could expect an increase of the normalized information distance. Furthermore, if the size of the window increases, the effect of the filtering increases as well and it is reasonable to assume that the NID becomes larger than the one for 3×3 window filter.

It should be noticed that there are algorithms that are not invertible at all. An example is the median filtering. For such algorithms, the information distance becomes:

(6) $$E\left(f,f^{*}\right)=\max\{|A|,K\left(R\left(f,f^{*}\right)\right).\}$$

We have investigated the median filtering for *Lena* in a 3×3 window, and we found 3.58 bits for the entropy of the residual, a larger value than for the case of moving average filtering inversion.

A typical case that can also yield large values for the information distances is the case of images corrupted by noise. The approximate inverse should be provided by a denoising algorithm, $A^{-1}$ plus some residual image. If one denotes the noise by η, A(f)=f+η one can have the information distance for the case of noisy images as:

(7) $$E\left(f,f^{*}\right)=\max\{K\left(\eta \right),|A^{-1}\left|+K\left(R\left(f,A^{-1}\left(f^{*}\right)\right)\right).\right\}$$

The above equation suggests that the information distance and, consequently, the normalized information distance can increase considerably with the noise.

Our analysis advocates that, in theory, the information distance between two versions of the same image, an original and a processed version, can take rather large values depending on the image processing algorithm. More precisely, even for simple image processing tasks (e.g., moving average or median smoothing), the NID can take large values, preventing the detection of the similarity between the two images. On the other hand, in practice, one measures the complexity of the image processing algorithm indirectly, by the size of the joint lossless compression of the original image and the processed version. This should introduce additional difficulties. For instance, if we consider two images and their low-pass versions, it is expected to compress more efficiently the joint image of the two low-pass versions than the joint original image and corresponding filtered version. In other words, NCD (Equation (3)) would detect similarity between the filtered versions of two different images, not between each original and its filtered version.

### 2.2. Vectors of Compression Distances

We have shown above that the theoretical model of image similarity detection by information distances has the drawback that, even for simple processing algorithms, because of the apparition of noise terms, the Kolmogorov complexity is not always negligible. The major drawback, however, is the implementation of information distances by using compression, since, instead of the true complexity, one has values depending on the joint compression of the images under consideration. Finally, the decision is based on a single value, the compression based distance between two images. Next, we propose a framework for the detection of image similarity more robust to common image processing. Instead of a single value, we shall use significantly more values (e.g., hundreds). The detection will not depend on the exact value of the complexity of the algorithm, but on the error provided by implementing the computation of information distance with a standard image compressor. Otherwise stated, the major drawback of the direct distance computation, the use of compression size for information distance computation, is the ground of the proposed method.

Our approach starts from the observation that, given two images *f* and *g* and their processed versions by considering the same algorithm, $f^{*}$ and $g^{*}$, one should have $E\left(f,f^{*}\right)=E\left(g,g^{*}\right)$. Similarly, if one considers *n* distinct processing algorithms and one computes the information distances between each image and its processed version, one should obtain two identical vectors of distances. If not, the errors are due to the compressor and to the content of the particular images considered in the experiment. Otherwise stated, each vector provides not only information on the processing algorithms, but also on the compressor and on the particular image content.

We shall elaborate on this idea. Instead of different image processing algorithms, we shall consider a reversible algorithm *T* depending on a parameter *i* with the particularity that the complexity of $T_{i}$ is independent of *i*. We shall further consider the sequence of image transforms:

(8) $$f_{i}=T_{i}\left(f\right),\;i=1,\ldots ,t.$$

A simple example of such transforms is the circular translation of the image. Thus, for the sake of simplicity, let *f* be a square gray-level image of size N×N and let us consider circular translations on vertical direction (rows). Let $T_{i}$ be the circular translation with *i* rows and let $f_{i}=T_{i}\left(f\right)$, with i=1,…,N. One has $f_{N}=f$. The complexity of the circular translation does not depend on the translation shift. Furthermore, *T* is invertible, the inverse is simply the shift in the opposite direction and one has $|T_{i}\left|=\right|T_{-i}|$. To conclude, the information distances between the original and the translated versions are all equal. It is also reasonable to assume $K\left(f\right)\approx K\left(f_{i}\right)$, ∀i, and, consequently, the sequence of normalized information distances, $NID(f,T_{i}\left(f\right))$ are also equal.

In practice, we approximate the NID with compression based distances like, for instance, the NCD of Equation (3). Given the image *f*, we compute the vector:

(9) $$D\left(f\right)=\left\{d_{1},d_{2},\ldots ,d_{N},\right\}$$

with

(10) $$d_{i}=NCD\left(f,T_{i}\left(f\right)\right).$$

The components $d_{i}$ depend on the transform *T*, the compressor used by the NCD and, of course, on the image content. Obviously, given an image *f*, it is very unlikely to have identical components of D(f), like for the theoretical case of NID. Our expectation is that the variation of the components of D(f) provides enough information on image content that can be further used for detecting image similarity. More precisely, if $f^{*}$ is a version of *f*, we expect that the components of $D(f^{*})$ and D(f) have a rather similar variation, i.e., we investigate not the exact values, but the shape of the vectors.

In conclusion, instead of computing compression based distances between two images to detect similarity, we consider for each image a vector of compression based distances between the original and a sequence of transformed versions, and we compare the two vectors. The transform and the compression based distance should provide a description of the 2D image content by means of the behavior of the image at compression. In the next two sections, we investigate the selection of transforms and compressors for practical implementation of our approach.

## 3. A Simple Experiment

The definition provided by Equations (8)–(10) is based on two elements, the sequence of transforms and the compression distance. Since the transform is meant to allow the extraction of 2D information about images, a natural candidate are geometric transforms like translations and rotations. We shall further consider five simple transforms: circular shift with *i* columns ($T_{i}^{h}$, horizontal direction), circular shift with *i* rows ($T_{i}^{v}$, vertical direction), circular shift with *i* of both columns and rows in diagonal direction ($T_{i}^{d}$), circular shift in inverse diagonal direction ($T_{i}^{id}$, 135${}^{\circ }$), rotation around the center of the image by iϕ degrees, counterclockwise, $T_{i}^{\phi }$. Before rotation, image is periodically extended both horizontally and vertically. After rotation, the central part is cropped.

The transforms are designed to preserve the size of the image (number of rows and columns). They are of very low complexity and, since we should compress the joint original and transformed images, an interesting aspect is the fact that the image first order statistics (histogram) is preserved.

Compression results for images can vary significantly and, consequently, the compression based distances vary accordingly. As also done in [15], we shall consider different compressors, both general purpose ones (WINZIP, WINRAR, GZIP, 7Z, FPAQ0f, LPAQ1, PAQ8, PAQ9) and image dedicated (JPEG, JPEG2000) or, equivalently, image representation formats with lossless compression (PNG, TIF).

The implementations for JPEG2000, JPEG, PNG, TIFF, and GZIP are the ones provided by Matlab. We use WinZiP version 19.5, the WinRar version 5.2.1 and the 7z version 9.38 beta. Finally, the compressors PAQ8, PAQ9, LPAQ1 and FPAQ0f are available at [26].

Equation (10) needs the compression of the joint *x* and *y*. When dealing with images, the usual solution is to convert them into 1D sequences, to concatenate the sequences and finally, to compress the concatenated sequences. Since, in our case, one has images of the same size, one can directly concatenate the two images by producing a single image either two times wider, or two times higher. One shall further consider both methods, namely the concatenation on rows (horizontal concatenation) and on columns (vertical concatenation).

By using the approach proposed in Section 2.2, one can generate, for each compressor, 10 distinct vectors (five transforms × 2 concatenation methods). In order to eliminate the configurations with low robustness, we shall consider a simple experiment of image classifications after standard median and moving average filtering. The configurations that cannot provide correct results are eliminated.

The correct classification means that the vectors preserve after filtering a good representation of the original image content. A similar definition of robustness is used in watermarking, namely as the ability of watermarks to survive signal processing operations [27]. Tools for robustness testing of image watermarking algorithms have been developed (see, for instance, StirMark [28]). In Section 3 and Section 4, we shall consider common image/signal processing and image manipulation tasks inspired by the ones of StirMark.

Let us consider two well-known standard gray-level test images of size 512×512, (*Lena* and *Barbara*). For each image, we generate two other versions: one by median filtering in a 3×3 window, another by moving average filtering in the same window. The PSNR for median filtering is 35.44 dB (*Lena*) and 25.43 dB (*Barbara*). For moving average, one has 25.43 dB (*Lena*) and 25.01 dB (*Barbara*). Both filters are used for noise smoothing. While the output of the median filter is one of the pixels within the filter window, the output of the moving average does not necessarily belong to the window (it is the average within the filter window).

For each transform, concatenation method and compressor, we generate the six distance vectors corresponding to the six test images. The size of the vectors depends on the length of the sequences of transforms. For translations, we consider a step of one row for vertical translations, one column for horizontal translations and both one row and one column for diagonal translations. Hence, the size of the corresponding feature vectors is 512 samples. For rotations, we consider an angle ϕ of two degrees that provides 180 samples.

Let the distance vectors of the original images be the prototypes of the two image classes and let us consider the Pearson’s correlation coefficient, *r*, to evaluate the similarity between a sample and the classes prototypes. A great advantage of using Pearson’s correlation coefficient is its invariance to linear transforms (S→aS+b, with a>0), i.e., one recovers the “shape” regardless of an offset or of the multiplication (scaling) with a positive constant.

In order to provide a preliminary evaluation of our approach, we consider the number of filtered versions correctly classified and the interclass distance. If the four filtered images are correctly classified and the difference between the interclass distance is greater than 0.1, the distance vector is labeled as *promising* (P), i.e., one can expect some robustness. If, even for this simple experiment, there are classification errors, one should expect *low* robustness (L). Finally, if the classification completely fails or the interclass distance is too low, the signature is bad (B). The results are presented in Table 1.

From Table 1, one can see that the vectors based on JPEG2000 provide promising results in six cases out of ten. The ones based on JPEG do not provide promising results.

**Figure 1:**
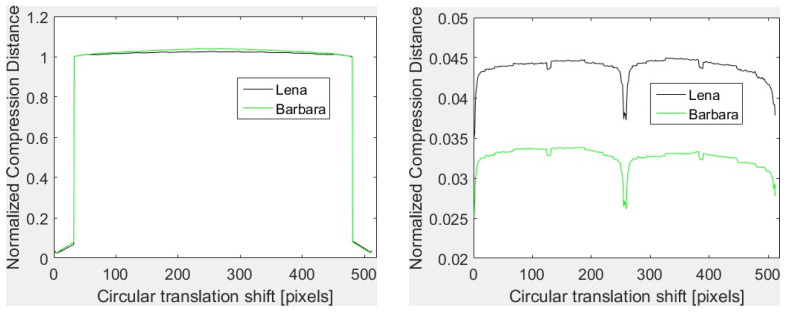
GZIP based vectors for *Lena* and *Barbara* with horizontal translation (**left**) and vertical translation (**right**).

Furthermore, the dictionary based compressors (like the ones based on LZ77, LZW) give rather poor results with translation transforms. This is the case of GZIP, WinRAR, WinZIP, 7Z and of the image formats with lossless compression like PNG and GIF. The basic principle of dictionary coding is to search the matches between the sequence to be compressed and a set of strings stored in a dictionary structure created during the encoding. The matched strings are substituted with references to the dictionary. Thus, the joint compression of the original and the translated version may depend not on the content of the image, but on the shift and on the coding of the references to the dictionary. Such an example is provided in Figure 1 for GZIP with $T^{h}$ and $T^{v}$ with horizontal concatenation. As it can be seen, in both cases, the shapes of the vectors are almost identical. In fact, for $T^{h}$ and low translation shifts (*i* and 512−i, with i≤32), the GZIP compressor detects the duplication of the data due to the shifts and the compression is very efficient. One has the size of the joint image almost equal to the size of a single image and, consequently, NCD is almost zero. As soon as *i* increases, the data duplication is no more detected, the size of the compressed joint image becomes two times the size of a single image and NCD takes values around one. For vertical translations, data duplication is detected. Thus, NCD takes values close to zero and the shapes are identical. To conclude, the vectors depend on the shift, not on the image content.

The PAQ compressors (PAQ8, PAQ9) provide rather promising results. These compressors are known as very efficient, but very slow. LPAQ1 and FPAQ0f give promising results only in two cases out of 10. Compared with the other PAQ versions, FPAQ0f (a simple order-0 arithmetic file compressor for stationary sources [29]) is considerably faster.

### Direct NCD Based Classification

Finally, we have also investigated the direct use of NCD in the simple classification experiment considered above. The 12 compressors are tested together with the two concatenation modes. The prototypes are the original images *Lena* and *Barbara*. For each filtered image, the NCD of each prototype is computed. The results are bad (Table 1). More precisely, in each case, we have found distances very close to 1. This means that the size of the joint compressed image is very close to the sum of the sizes of the two compressed images.

For instance, let us briefly investigate some results for JPEG2000 with horizontal concatenation. The NCD between the moving average filtered *Lena* and the prototypes *Lena* and *Barbara* are 0.9994 and 1.0009. The ones for moving average filtered *Barbara* and orginals *Lena*, *Barbara* are 0.9990 and 0.9995, respectively. With such small differences between distances, one cannot consider that the filtered *Lena* version is correctly classified and the *Barbara* one is not. In addition, the distance between the originals is 1.0006 and the one between the moving average versions is 0.9998. As we said above, the results are very similar, regardless of compressor, concatenation mode or image pair.

To conclude, for some distance vectors based on NCD, one gets correct classification for the mild filtering experiment and, implicitly, one can expect some robustness. For the direct use of NCD, the classification completely fails.

## 4. Robustness: A Case Study

**Figure 2:**
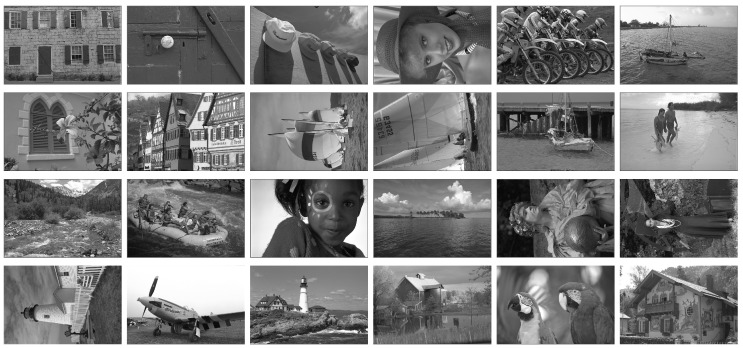
Gray-level versions of Kodak test images 1–24: from left to right and from top to bottom.

The promising configurations determined in Section 3 are next evaluated by testing their robustness on the gray-level version of the Kodak data set (Figure 2). The Kodak set is composed of 24 true color (24 bits) images of sizes 512×768 (available at [30]). We consider the gray-level versions of the full color Kodak test images.

While *Lena* and *Barbara* are square images of sizes 512×512, the Kodak test images are rectangular of sizes 512×768 and 768×512. Thus, with translation, one can have either 512 or 768 samples. In order to compare the vectors, a simple solution is the transposition of the 768×512 images. Another solution is the resizing before the computation of the correlation coefficient. We shall further consider the results for image transposition.

The robustness of a selected configuration (compressor, transform and concatenation method) against a given image processing task (histogram equalization, smoothing, lossy compression, etc.) is evaluated as follows. The feature vectors for the original gray-level Kodak test images are considered the prototypes of the 24 classes. The processed images are assigned according to the maximum of the correlation coefficient between their feature vectors and classes prototypes. The fraction of correctly classified processed images provides information on the robustness of the selected signature against the considered image processing task.

### 4.1. Robustness against Histogram Equalization

We consider first the robustness against histogram equalization. While from Section 2.1 it follows that the theoretical information distances between original and equalized images is lower than between original and moving average or median filtered images, in practice, the distortion introduced by histogram equalization is considerably more severe than the one introduced by filtering in 3×3 windows. Thus, the PSNR of the equalized Kodak images ranges between 10.31 dB (*Kodim02*) and 22.00 dB (*Kodim08*), while the one of moving average filtered images ranges between 22.84 dB (*Kodim08*) and 33.17 dB (*Kodim23*). The distortion for moving average is slightly lower than the one of median filtering, namely the PSNR ranges between 23.84 dB (*Kodim13*) and 36.57 dB (*Kodim23*).

The correct classification rates for the promising candidates selected in Section 3 are as follows:

- JPEG2000: 21/24 for horizontal concatenation with $T^{v}$, 20/24 for vertical concatenation with $T^{id}$, 18/24 for both horizontal and vertical concatenation with rotation, 17/24 for vertical concatenation with both horizontal and diagonal translation;
- GZIP: 8/24 for vertical concatenation with rotation;
- WinZIP: 19/24 for vertical concatenation with $T^{v}$, 15/24 for horizontal concatenation with $T^{v}$ and 8/24 for vertical concatenation with $T^{h}$;
- WinRAR: 14/24 for horizontal concatenation with rotation;
- 7Z: 8/24 for vertical concatenation with $T^{d}$, 6/24 for vertical concatenation with $T^{h}$;
- PAQ8: 19/24 for vertical concatenation with $T^{h}$, 14/24 for vertical concatenation with $T^{d}$ and $T^{id}$, only 7/24 for horizontal concatenation with $T^{h}$
- PAQ9: 20/24 for horizontal concatenation with $T^{v}$, 17/24 for vertical concatenation with $T^{v}$, 15/24 for horizontal concatenation with $T^{h}$, 8/24 for horizontal concatenation with $T^{h}$;
- LPAQ1: 17/24 for horizontal concatenation with $T^{h}$ and 15/24 for horizontal concatenation with $T^{v}$;
- FPAQ0f: 24/24 for horizontal concatenation with rotation; 23/24 for horizontal concatenation with $T^{v}$.

From the above-mentioned results, it clearly appears that the correct classification for two classes does not guarantee good results for 24 classes. On the other hand, very good results are provided for the two configurations with FPAQ0f (the one with rotation correctly classifies all the 24 equalized images). Good results are also provided by the configurations based on JPEG2000 and for PAQ9 (horizontal concatenation with $T^{v})$. As somehow expected, the signatures using dictionary based compression provide rather poor results regardless of the geometric transform. An exception is offered by WinZIP (vertical concatenation, vertical translation) that provides 19 correct results. The configurations (compressor, transform and concatenation) that provides at least 20/24 correct classification results (i.e., more than 80%) are listed in Table 2. We shall continue the robustness tests only for two configurations out of the four, FPAQ0f with horizontal concatenation and rotation, JPEG2000 with horizontal concatenation and vertical translation.

### 4.2. More Results on Robustness

Next, we investigate the robustness of the two selected vectors against smoothing, noise, JPEG lossy compression, exact histogram equalization as well as against some geometric transforms (scaling, cropping and rotation). The correct classification results are presented in Table 3.

We consider first the smoothing by linear filtering (moving average and Gaussian filtering) and by nonlinear filtering (median). For each filter, three sizes of windows have been used, 3×3, 5×5 and 7×7. The robustness is good for filtering with 3×3 window, slightly better for FPAQ0f. Thus, one gets 22/24 (moving average), 24/24 (Gaussian filtering), 23/24 (median filtering) for FPAQ0f and 21/24 (moving average), 23/24 (Gaussian filtering), 22/24 (median filtering) for JPEG2000. As expected, the correct classification rate decreases with the window size (see Table 3).

**Figure 3:**
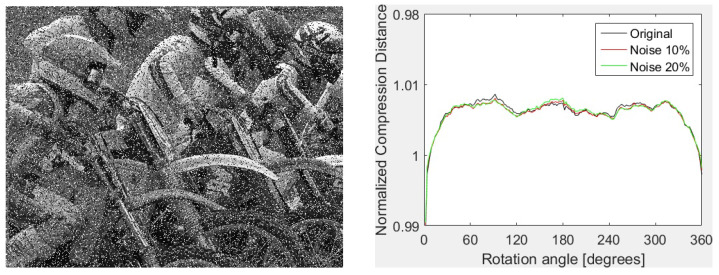
Details of test image *Kodim5* corrupted with 20% “salt and paper” noise (**left**) and sequence of FPAQ0f signatures for the original, and two noisy versions with 10% and 20% (**right**).

The proposed feature vectors provide some robustness against noise as well. Both configurations give very good results at a low level of noise ($\sigma^{2}=0.0001$ for Gaussian and density of 0.01% for “salt and pepper” noise). Furthermore, the remarkable robustness of FPAQ0f vectors against “salt and pepper” noise should be noticed. For instance, correct classification (24/24) is also obtained for 20% noise density. An example of image corrupted with 20% “salt and pepper” noise (left) and the shape of FPAQ0f vectors for the original image and two copies corrupted with “salt and paper” noise of 10% and 20% density are presented in Figure 3. As one can see, the shapes are almost indistinguishable.

The results show robustness against JPEG lossy compression, too. For QF ≥80, the 24 compressed images are correctly classified. The results are still good for QF=40, namely 21 (FPAQ0f) and 22 (JPEG2000) images are still correctly assigned.

Besides the classical equalization by cumulative distribution function, we consider also the exact histogram equalization [24]. The exact equalization provides the same results as the classical one: 24/24 for FPAQ0f and 21/24 for JPEG2000.

We investigate next the robustness against some simple geometric transforms, namely scaling, cropping and rotation. Both upscaling and downscaling is done by using Matlab function *resample* with bicubic interpolation. As one can see from Table 3, FPAQ0f provides very good results for all the scale factors (no errors except for downscaling with $\frac{1}{4}$ (2 errors) and $\frac{1}{2}$ (1 error)). JPEG2000 provides good results only for the scale factors $\frac{1}{2}$ and 2.

We also tested the robustness against cropping of the *n* first/last rows and columns of the image. FPAQ0f provides a rather good robustness against cropping, for instance, 24/24 for n=16 and 23/24 n=32 (for n=32, only 80% of the image is preserved). On the contrary, JPEG2000 provides no robustness against cropping. We tested also the robustness against rotation with respect to image center. As one can see, FPAQ0f exhibits some robustness.

**Figure 4:**
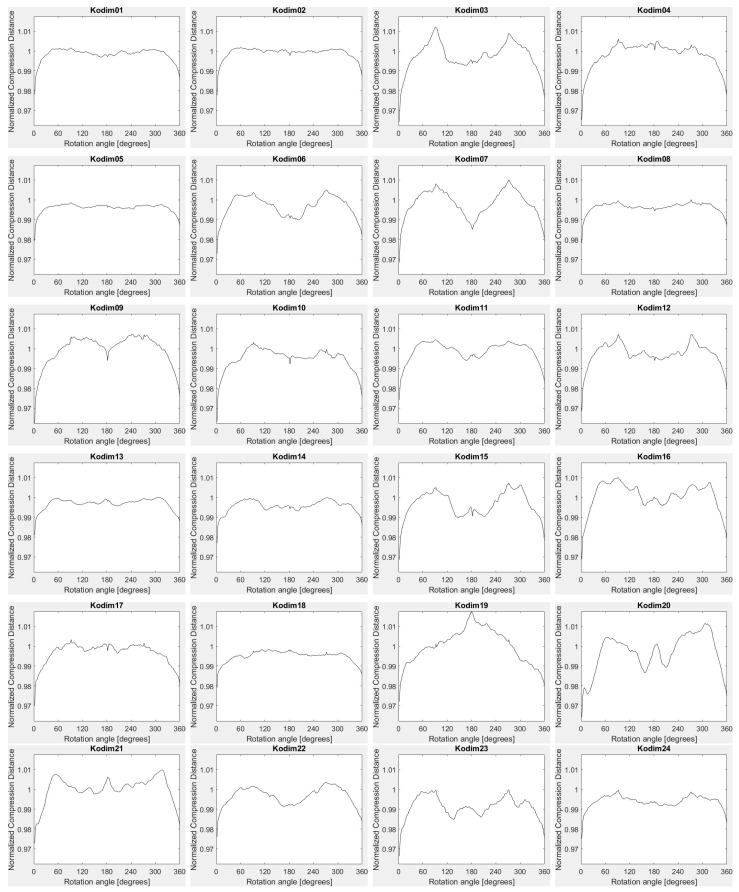
*Kodim01*–*Kodim24*: feature vectors for PAQ0f and rotation.

To conclude, for smoothing, noise, JPEG lossy compression and exact histogram equalization both configurations provide rather similar results. The configuration based on FPAQ0f provides some robustness against geometric attacks, as well. The vectors based on FPAQ0f also have the advantage that they are independent of the size of the image. Thus, the distance between vectors can be directly computed, without any resizing. The shape of the vectors with FPAQ0f for Kodak test images is shown in Figure 4.

**Figure 5:**
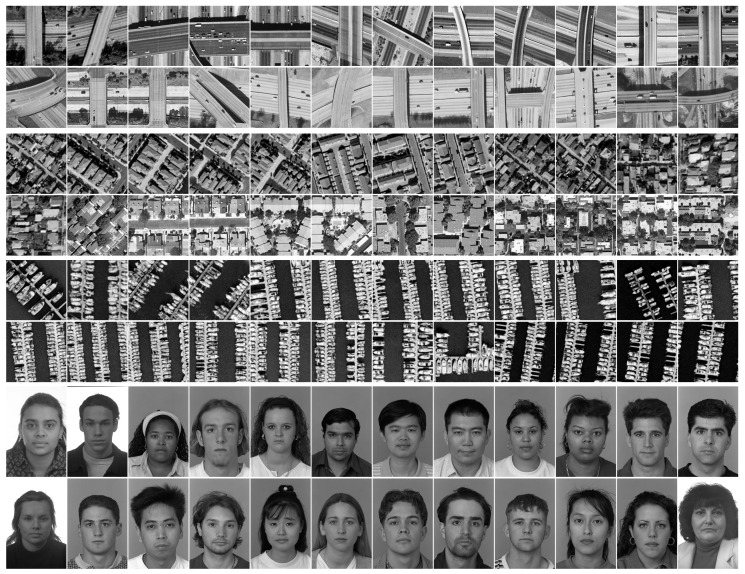
Four data sets: *Overpass* (rows 1–2); *Denseresidential* (rows 3–4); *Harbor* (rows 5–6); and *Faces* (rows 7–8).

The results presented so far have been obtained on a set of 24 high quality natural images representing very different scenes. The intrinsic heterogeneity of the image set is not essential for the robustness at similarity detection in the case of common image processing. In this regard, we also consider more homogeneous test sets composed of images grouped according to their content, and we investigate the robustness against histogram equalization of the two selected configurations. The four sets used in this experiment are displayed in Figure 5. Three sets are composed of 256×256 images from UC Merced Land Use Dataset, [31,32], namely the first 24 images of the classes *Overpass*, *Denseresidential* and *Harbour*. The fourth set is composed of 24 facial images of size 384×256 from Face Recognition Technology (FERET) database (available at [33]). The correct classification rates for the configuration based on FPAQ0f are as follows: 24/24 on *Denseresidential* and *Harbor*, 23/24 on *Overpass* and 22/24 on *Faces*. The results for JPEG2000 based feature vectors are 21/24 on *Overpass*, 17/24 on *Denseresidential*, 14/24 on *Harbor* and 13/24 on *Faces*. As one can see, the selected configurations exhibit robustness on homogeneous data sets as well. The results are rather similar with the ones of Section 4.1.

### 4.3. Robustness and Mathematical Complexity

The robustness of feature vectors based on compression distance comes at the cost of computational complexity. Instead of a single compression distance, one should compute a sequence of distances. The computational complexity depends on the size of the transforms. The results presented above have been obtained for large sequences of transforms (rotations of $2^{\circ }$ and translations with one row/column). The size of the sequences of transforms can be considerably decreased without major impact on the robustness of the signatures.

Thus, we have considered the histogram equalization experiment for the case of FPAQ0f compressor with rotations with larger angles, i.e., for shorter sequences of transforms. Thus, we have tested the robustness of vectors with 45 components ($8^{\circ }$ rotations). The results are very similar with the ones obtained for 180 components. Thus, one gets exactly the same results at smoothing, lossy compression and exact histogram equalization. For robustness against noise, there are some very small differences. The length of the transform can be decreased for JPEG vectors as well. For instance, for 128 samples instead of 512, the correct classification obtained for JPEG2000 vectors is 20/24 instead of 21/24.

## 5. Conclusions

Feature vectors of compression distances between original image and a sequence of simple geometric transforms have been proposed and their usefulness in similarity detection in the case of common image processing has been discussed. Several compressors, transforms (translations on principal directions and rotations) and concatenation modes (for the computation of compression distance) have been investigated. While the direct use of the NCD fails for detecting similarity even in the simple case of two images and their median and moving average filtered versions in 3×3 windows, the proposed approach provides, for some configurations, promising results. Two configurations, FPAQ0f compression, rotation transform, horizontal concatenation and JPEG2000, vertical translation, and horizontal concatenation appeared to provide robustness against smoothing, lossy compression, contrast enhancement, and noise addition in an experiment on 24 images (the gray-level versions of Kodak set). The first one also provides some robustness against geometrical transforms.

## Figures and Tables

**Table 1:** Classification results: P (promising), L (low robustness) and B (bad robustness).

	Horizontal Concatenation					Vertical Concatenation					NCD	
	$T^{h}$	$T^{v}$	$T^{d}$	$T^{\mathrm{id}}$	$T^{\phi }$	$T^{h}$	$T^{v}$	$T^{d}$	$T^{\mathrm{id}}$	$T^{\phi }$	H	V
JPEG2000	L	P	L	L	P	P	L	P	P	P	B	B
JPEG	L	L	L	L	L	L	L	L	L	L	B	B
PNG	B	B	B	B	B	B	L	B	B	L	B	B
TIFF	B	B	B	B	B	B	B	B	B	L	B	B
GZIP	B	B	B	B	B	B	L	B	B	P	B	B
WinZIP	L	P	L	L	B	P	P	L	L	B	B	B
WinRAR	L	L	L	L	P	L	L	L	L	P	B	B
7Z	L	L	L	L	L	L	P	L	P	B	B	B
PAQ8	P	L	L	L	B	P	L	P	P	L	B	B
PAQ9	P	P	L	L	B	P	P	L	L	B	B	B
LPAQ1	P	L	L	L	B	P	L	L	L	B	B	B
FPAQ0f	L	P	L	L	P	L	L	L	L	L	B	B

**Table 2:** Configurations with at least 20/24 correct classification results for histogram equalization.

Compressor	Transform	Concatenation	Results
FPAQ0f	Rotation	Horizontal	24/24
FPAQ0f	Rotation	Vertical	23/24
JPEG2000	Vertical translation	Horizontal	21/24
JPEG2000	Inverse diagonal	Vertical	20/24

**Table 3:** More results for the selected configurations on Kodak set.

		FPAQ0f			JPEG2000		
Mooving average	Window size	3×3	5×5	7×7	3×3	5×5	7×7
	Results	22/24	18/24	17/24	21/24	21/24	19/24
Gaussian filtering	Window size	3×3	5×5	7×7	3×3	5×5	7×7
	Results	24/24	20/24	18/24	23/24	22/24	20/24
Median filtering	Window size	3×3	5×5	7×7	3×3	5×5	7×7
	Results	23/24	21/24	18/24	22/24	18/24	16/24
Gaussian noise	$\sigma^{2}$	0.0001	0.0005	0.001	0.0001	0.0005	0.001
	Results	24/24	21/24	21/24	24/24	19/24	19/24
“Salt and pepper” noise	Density	0.01%	0.05%	0.1%	0.01%	0.05%	0.1%
	Results	24/24	24/24	24/24	24/24	17/24	11/24
Lossy compression	Quality (QF)	80	40	20	80	40	20
	Results	24/22	22/24	18/24	24/24	22/24	20/24
Downscaling	Scale factor	3/4	1/2	1/4	3/4	1/2	1/4
	Results	24/24	23/24	22/24	2/24	23/24	18/24
Upscaling	Scale factor	3/2	7/4	2	3/2	7/4	2
	Results	24/24	24/24	24/24	13/24	7/24	22/24
Cropping	Rows & columns	16	32	48	2	4	-
	Results	24/24	23/24	18/24	11/24	4/24	-
Rotation	Degrees	$2.5^{\circ }$	$5^{\circ }$	$7.5^{\circ }$	$2.5^{\circ }$	-	-
	Results	24/24	23/24	20/24	9/24	-	-
